# Invasive beta-haemolytic streptococcal infections, Finland, 2006 to 2020: increase in Lancefield group C/G infections

**DOI:** 10.2807/1560-7917.ES.2023.28.31.2200807

**Published:** 2023-08-03

**Authors:** Dafni Katerina Paspaliari, Emmi Sarvikivi, Jukka Ollgren, Jaana Vuopio

**Affiliations:** 1 Finnish Institute of Health and Welfare, Helsinki, Finland; 2 ECDC Fellowship Programme, Public Health Microbiology path (EUPHEM), European Centre for Disease Prevention and Control (ECDC), Solna, Sweden; 3 University of Turku, Institute of Biomedicine, Turku, Finland; 4 Turku University Hospital, Clinical Microbiology, Turku, Finland

**Keywords:** Streptococcus, GAS, GBS, GCS, GGS, streptococcal infections, Streptococcus pyogenes, Streptococcus agalactiae

## Abstract

**Background:**

Invasive infections with beta-haemolytic streptococci of Lancefield groups A (iGAS), B (iGBS) and C/G (iGCGS) are a major cause of morbidity and mortality worldwide.

**Aim:**

We studied incidence trends of invasive beta-haemolytic streptococcal infections in Finland, focusing on iGCGS.

**Methods:**

We conducted a retrospective register-based study. Cases were defined as isolations from blood and/or cerebrospinal fluid and retrieved from the National Infectious Disease Register where all invasive cases are mandatorily notified.

**Results:**

Between 2006 and 2020, the mean annual incidence was 4.1 per 100,000 for iGAS (range: 2.1–6.7), 5.2 for iGBS (4.0–6.3) and 10.1 for iGCGS (5.4–17.6). The incidence displayed an increasing trend for all groups, albeit for iGBS only for individuals 45 years and older. The increase was particularly sharp for iGCGS (8% annual relative increase). The incidence rate was higher in males for iGCGS (adjusted incidence rate ratio (IRR) = 1.6; 95% confidence interval (CI): 1.5–1.8) and iGAS (adjusted IRR = 1.3; 95% CI: 1.1–1.4); for iGBS, the association with sex was age-dependent. In adults, iGCGS incidence increased significantly with age. Recurrency was seen for iGCGS and secondarily iGBS, but not for iGAS. Infections with iGCGS and iGBS peaked in July and August.

**Conclusions:**

The incidence of invasive beta-haemolytic streptococcal infections in Finland has been rising since 2006, especially for iGCGS and among the elderly population. However, national surveillance still focuses on iGAS and iGBS, and European Union-wide surveillance is lacking. We recommend that surveillance of iGCGS be enhanced, including systematic collection and typing of isolates, to guide infection prevention strategies.

**Key public health message**

**What did you want to address in this study?**

Beta-haemolytic streptococci can cause severe invasive infections with considerable morbidity and mortality. In this study we wanted to investigate whether the incidence of this type of infections has changed over the 15-year period 2006 to 2020 in Finland. We additionally wanted to identify population groups with an increased risk for this type of infections.

**What have we learnt from this study?**

In Finland, over the period 2006 to 2020, there was an increase in the incidence of invasive infections caused by beta-haemolytic streptococci. The sharpest increase was observed for infections caused by streptococci belonging to Lancefield groups C/G, which mostly affected older age groups.

**What are the implications of your findings for public health?**

The observation of a steep rise in invasive infections caused by streptococci of groups C/G is of concern and warrants further investigation. To this end, we propose that the surveillance of this type of infections be enhanced.

## Introduction

Beta-haemolytic streptococci are important bacterial pathogens, known to cause considerable morbidity and mortality worldwide both in adults and in children [1-4].

Beta-haemolytic streptococci are traditionally classified into Lancefield serogroups [5]. The two groups best known and characterised in terms of pathogenicity and burden of disease are group A (GAS; *Streptococcus pyogenes*) and group B (GBS; *S. agalactiae*). Streptococci belonging to groups C (GCS) and G (GGS), predominantly of the species *S. dysgalactiae* subspecies *equisimilis*, can also cause human infections, and their importance is increasingly being acknowledged [6-8]. The species overlap within the Lancefield serogroups. For example, *S. dysgalactiae* subspecies *equisimilis* can exhibit group C, group G and also sometimes group A antigens. Besides Lancefield serotyping, *emm* typing can be used for molecular typing of GAS, GCS and GGS; it is based on polymorphisms in the *emm* gene that encodes the M protein, a major streptococcal virulence factor [9,10].

Infections caused by beta-haemolytic streptococci present with a wide clinical range, from mild superficial infections to severe life-threatening invasive diseases. Invasive GAS infections (iGAS) may be fulminant and cause considerable mortality. Typical clinical manifestations include bacteraemia, necrotising fasciitis and toxic shock syndrome. Although less frequent than non-invasive GAS diseases, their global burden is high, with 163,000 associated deaths per year based on a 2005 estimate [1]. Invasive infections with GBS (iGBS) manifest as bacteraemia and meningitis, primarily in newborn infants and the elderly population [2]. Invasive group C and group G infections (iGCGS) have clinical manifestations similar to iGAS infections, which has been attributed to the relatedness and similarities between *S. dysgalactiae* and *S. pyogenes* [10]. Asymptomatic carriage also occurs, especially for GAS and GBS.

Invasive streptococcal infections in Finland are mandatorily notified by the clinical microbiological laboratories to the National Infectious Disease Register (NIDR) of the Finnish Institute for Health and Welfare (THL) since 1995. All iGAS and iGBS isolates are sent to the reference laboratory of THL for surveillance purposes, with the possibility for typing. In contrast, iGCGS isolates are processed at the local laboratory level and not collected in a centralised manner, although iGCGS findings are also notifiable.

The aim of this study was to describe the changes in the incidence trends of invasive beta-haemolytic streptococcal infections in Finland over the 15-year period 2006 to 2020, with a focus on iGCGS, for which published studies at a national level are lacking. 

## Methods

### Notifications and definitions

An invasive beta-haemolytic streptococcal infection was defined as the isolation of beta-haemolytic streptococci from a blood or cerebrospinal fluid (CSF) specimen (NIDR case definition). In Finland, invasive beta-haemolytic streptococcal isolates are notified by local laboratories to the NIDR. Multiple notifications for the same individual are combined into a single case, when received within 3 months from first isolation.

Our study included all NIDR case notifications of isolations from blood or CSF with date of sampling between 1 January 2006 and 31 December 2020, with either of the following entries: group A streptococci, *S. pyogenes*, group B streptococci, *S. agalactiae*, group C streptococci, group G streptococci, *S*. *dysgalactiae* ssp. *equisimilis, S*. *dysgalactiae* ssp. *dysgalactiae*, *S*. *equi* ssp. *zooepidemicus*, *S*. *equi* ssp *equi*, *S*. *canis*. Case notification data included the national personal identification number, age, sex, specimen type, microbial species, Lancefield group and date of death. *Emm*-typing is not done as part of routine surveillance for iGCGS. As this information was lacking for most of these isolates, we decided not to include this variable in the analysis. Cases were dated based on the date of specimen collection or, in the few cases where that was not available, on the date when the notification was filed to the NIDR. Each calendar year spanned 1 January to 31 December.

The descriptive analysis included the calculation of interquartile ranges (IQR). Infants were defined as children younger than 1 year. Neonatal early-onset GBS disease was defined as occurring during the first 6 days of life, and late-onset between days 7 and 89. Multiple notifications for the same individual concerning the same Lancefield group were considered as repeat infections. The 7-day and 30-day case fatality ratios (CFR) were defined as the proportion of cases who passed away within, respectively, 7 and 30 days from the date of the case notification, which corresponded to the date of specimen collection in all but two cases.

### Analysis of incidence rates of iGAS, iGBS and iGCGS

To calculate incidence rates, mean population data for Finland, defined as the average of the populations of two consecutive years (the statistical year and the year before that) were retrieved from Statistics Finland. Age distributions between Lancefield groups were compared with a Wilcoxon rank sum test. To calculate trends in incidence rates while assessing their possible associations with age, sex and year of isolation, we used negative binomial regression. The need for interactions between explanatory variables in the model was assessed by Akaike information criterion or Bayesian information criterion. In all cases, there were age–sex interactions. For iGAS and iGCGS, we found no evidence of interaction between the annual trend and age or sex, except for a low-level interaction in ages 85 years and above. For iGBS, there were annual trend differences in several age groups, which we addressed by switching to a random (mixed) effect Poisson regression model.

With the aid of the models, we calculated incidence rate ratios (IRR), adjusted for the independent variables used in the respective models as further specified in Supplementary Tables S1 and S2. Seasonality was evaluated with Poisson regression, with year and month as independent variables, using monthly incidence data. We also ran the model with incidence data adjusted for the number of days per month. We calculated IRR per calendar year and month, adjusted for calendar month and calendar year, respectively.

All statistical analyses were carried out in Stata, version 17.0 (StataCorp LLC). P values below 5% or non-overlapping 95% confidence intervals (CI) were considered statistically significant. In cases of minimally overlapping 95% CIs and large differences in point values, statistical significance was confirmed with additional tests, where applicable.

## Results

### Incidence of invasive beta-haemolytic streptococcal infections 

**Figure 1:**
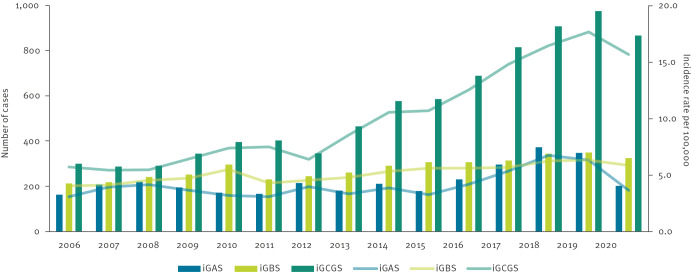
Annual number of cases and mean annual incidence rate per 100,000 of iGAS, iGBS and iGCGS, Finland, 2006–2020 (n =15,781)

Between 2006 and 2020, THL received a total of 15,781 notifications of invasive beta-haemolytic streptococcal findings: 15,663 (99.2%) from blood specimens, 29 (0.2%) from CSF and 89 (0.6%) from both. Of the notifications, 3,352 (21.2%) concerned iGAS, 4,190 (26.6%) iGBS and 8,239 (52.2%) iGCGS. On average, THL received 223 iGAS (range: 162–372), 279 iGBS (range: 213–350) and 549 iGCGS (range: 287–974) notifications annually. The respective mean annual incidence rate was 4.1 (iGAS), 5.2 (iGBS) and 10.1 (iGCGS) per 100,000 population. The annual incidence rate fluctuated over the years, ranging between 2.1 and 6.7 per 100,000 population for iGAS, between 4.0 and 6.3 per 100,000 population for iGBS and between 5.4 and 17.6 per 100,000 population for iGCGS, peaking in 2018 and 2019 (Figure 1).

iGAS: invasive group A *Streptococcus*; iGBS: invasive group B *Streptococcus*; iGCGS: invasive group C and G *Streptococcus*.

Columns show the case numbers, lines show the incidence rates.

Among the 512 neonatal cases of iGBS, 283 (55%) were early-onset and 229 (45%) late-onset. The mean annual incidence rate among infants was 63.0 per 100,000, in contrast to 4.6 per 100,000 for the rest of the population.

Over the 15-year period from 2006 to 2020, the mean annual incidence rate of invasive beta-haemolytic streptococcal infections showed an increasing trend for iGAS and iGCGS (Figure 1). The increase was particularly pronounced in the years 2018 and 2019, reaching 17.6 per 100,000 population in 2019 for iGCGS, before subsiding in 2020. We estimated the annual relative increase to be 3% for iGAS (adjusted IRR = 1.3; 95% CI: 1.1–1.4) and 8% for iGCGS (adjusted IRR = 1.6; 95% CI: 1.5–1.8). A detailed analysis of the annual relative IRR can be found in Table S1 in the Supplement. For iGBS, there were differences in the trends between age groups, with a significantly increasing trend among those 45 years and older, a stable or slightly decreasing trend among those aged 1–44 years, and a significantly decreasing trend among infants. Further details are appended in Supplementary Table S1.

### Distribution of cases by age and sex

**Figure 2:**
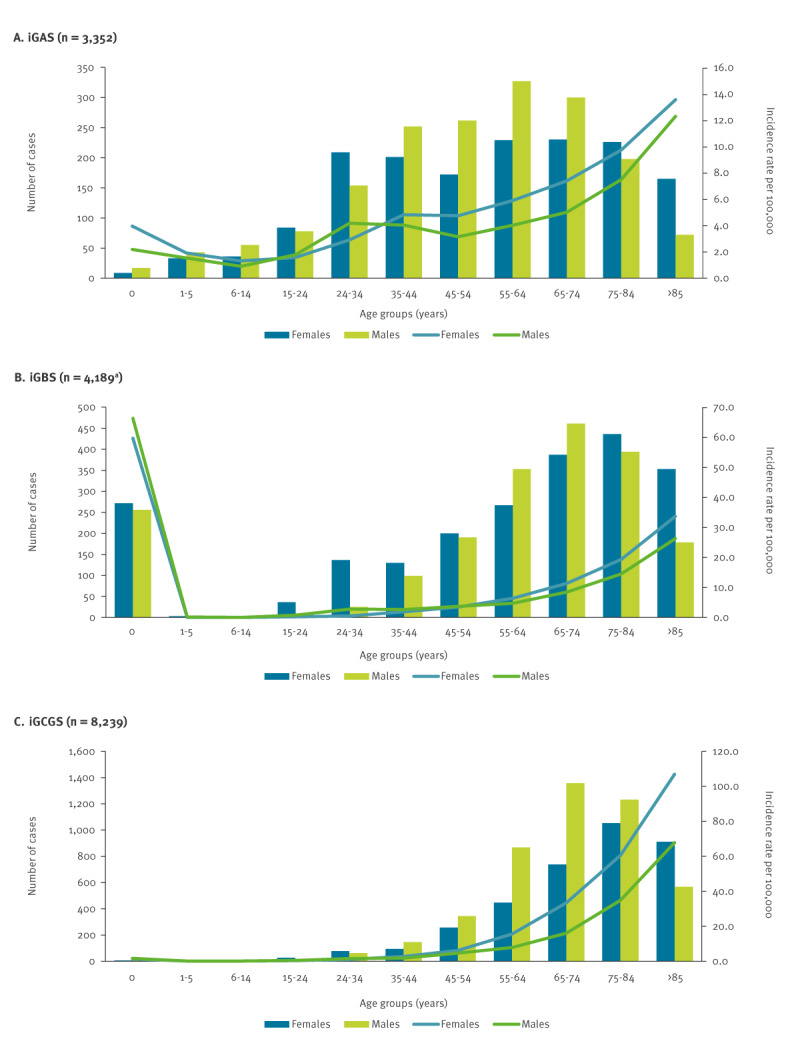
Number of cases and mean annual incidence rate per 100,000 of iGAS, iGBS and iGCGS infections per age group, for males and females, Finland, 2006–2020 (n = 15,780^a^)

The age–sex distribution of cases differed between the three Lancefield groups (Table 1, Figure 2). The median age of cases was significantly lower for iGAS (56 years) than for iGBS (66 years) and iGCGS (73 years). In addition, with the exception of infants, the incidence of iGAS was higher than that of iGBS and iGCGS in children younger than 15 years (Figure 2). In contrast, the incidence among infants was highest for iGBS.

**Table 1:** Age and sex characteristics of cases of iGAS, iGBS and iGCGS, Finland, 2006–2020 (n =15,781^a^)

Group	Male (n)	Female (n)	Male (%)	Female (%)	Median age (years)	IQR (years)
iGAS	1,758	1,594	52	48	56	37–71
iGBS all ages	1,968	2,221	47	53	66	47–78
iGBS age ≥ 1year	1,712	1,949	47	53	69	56–80
iGCGS	4,615	3,624	56	44	73	62–82

iGAS: invasive group A *Streptococcus*; iGBS: invasive group B *Streptococcus*; iGCGS: invasive group C and G *Streptococcus*; IQR: interquartile range.

^a^ Sex was unknown in one case, but this case is included in the columns on age.

iGAS: invasive group A *Streptococcus*; iGBS: invasive group B *Streptococcus*; iGCGS: invasive group C and G *Streptococcus*.

^a^ One neonatal case is excluded here because sex was unknown.

Columns show the case numbers, lines show the incidence rates.

With the exception of infant iGBS cases, the incidence rate increased with age for all three Lancefield groups; however, the level of increase differed, with iGCGS cases showing a marked age-specific increase, especially for ages of 55 years and above (Figure 2). The adjusted IRR per age group and sex are listed in Supplementary Table S2. The association with age was stronger for males than females (Figure 2). 

With regards to sex, there were more male cases of iGAS and iGCGS, and more female cases of iGBS (Table 1). The incidence rate was overall higher in males than females for iGCGS (adjusted IRR = 1.6; 95% CI: 1.5–1.8) and iGAS (adjusted IRR = 1.3; 95% CI: 1.1–1.4); for iGBS, the association was age group-dependent; for a detailed list of the respective IRR see Supplementary Table S2. For iGAS, male sex was positively associated with incidence among those aged 45–74 years, with a potential weaker association for those aged 75–84 years; we append these numbers in Supplementary Table S2. No association with sex was found for the younger age groups. Similarly, for iGCGS, the incidence rate was significantly higher in males than in females for those 35 years and older, but not for younger individuals. For iGBS, the association with sex was more complex and age-dependent. For example, for infants there was no difference between the sexes, whereas for the age group 25–44 years, female sex was a risk factor for iGBS infections. Conversely, male sex was a risk factor among those 55 years and older.

### Repeat infections

All cases of repeat infection with the same Lancefield group are presented in Table 2.

**Table 2:** Notified cases of iGAS, iGBS and iGCGS, and number and percentage of individuals with one vs multiple case notifications, Finland, 2006–2020 (n = 15,781)

	Notified cases	Number of individuals	Number of individuals with			
			One episode		Multiple episodes	
			n	%	n	%
iGAS	3,352	3,324	3,296	99.2	28	0.8
2 notifications					28	0.8
iGBS	4,190	4,037	3,906	96.8	131	3.2
2 notifications					112	2.8
3–4 notifications					19	0.5
iGCGS	8,239	7,668	7,201	93.9	467	6.1
2 notifications					406	5.3
3 notifications					51	0.7
4–6 notifications					10	0.1

iGAS: invasive group A *Streptococcus*; iGBS: invasive group B *Streptococcus*; iGCGS: invasive group C and G *Streptococcus*.

For iGAS, repeat infections were rare; the vast majority of individuals (> 99%) presented with a single episode of invasive infection. The maximum number of notifications for the same individual was two. The median interval between first and second episode was 404 days (range: 92–2,404); however, this result should be interpreted with caution, because the number of iGAS repeat infections was small.

Repeat infections were more common for iGBS (ca 3% of the individuals), with up to four notifications for the same individual. Sixty-four per cent of the individuals with repeat notifications were female. There were no cases of repeat infection among infants; the median age of the individuals at second infection was 68 years (IQR: 59–78) and the median interval between first and second episode was 455 days (range: 91–2,603).

Repeat infections were most common for iGCGS, affecting more than 6% of the iGCGS-infected individuals. The multitude of episodes was up to six episodes for the same individual. Of the cases with repeat notifications, 56% were male. The median age upon the second and third notified infection episode was 73 and 74 years, respectively (IQR for second episode: 63–81 years) and the median interval between first and second episode was 481 days (range: 92–3,879).

### Case fatality ratios

There were no deaths caused by iGAS or iGCGS among infants. In contrast, there were six deaths among infant cases with iGBS (median age of fatalities: 8 days; range: 0–34 days). All deaths occurred within 7 days from sampling, corresponding to a 7-day CFR of 1.1% for this age group. The 7-day and 30-day CFR for those aged ≥ 1 year are shown in Table 3.

**Table 3:** All-cause 7-day and 30-day case fatality ratios associated with iGAS, iGBS and iGCGS infections, for ages ≥ 1 year, Finland, 2006–2020 (n = 789 and 1,404, respectively)

Group	Deaths (n)	CFR (%)	Median age (years)	Age range (years)
7-day CFR				
iGAS	220	6.6	64	2–98
iGBS	161	4.4	76	21–102
iGCGS	408	5.0	78	30–102
30-day CFR				
iGAS	326	9.8	70	2–98
iGBS	309	8.4	78	21–102
iGCGS	769	9.3	79	30–102

CFR: case fatality ratio; iGAS: invasive group A *Streptococcus*; iGBS: invasive group B *Streptococcus*; iGCGS: invasive group C and G *Streptococcus.*

The median age and age range of the fatalities at the time of death are also shown.

### Seasonality

**Figure 3:**
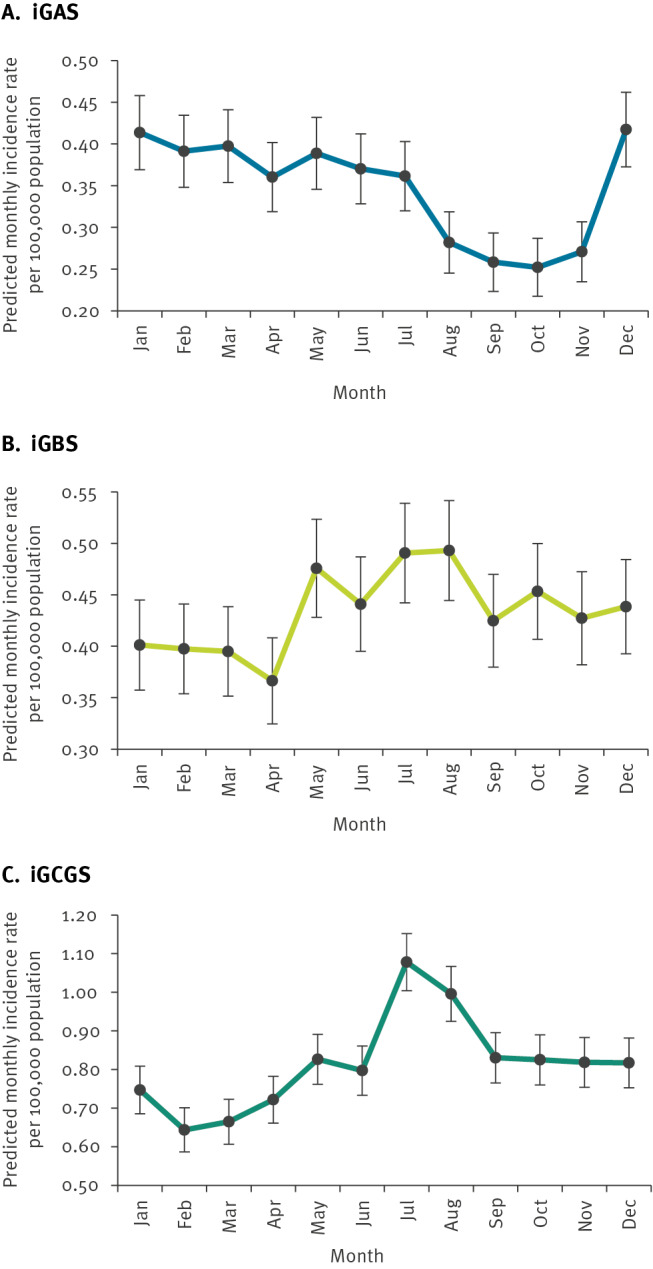
Monthly incidence rate per 100,000 population of iGAS, iGBS and iGCGS, as predicted by a Poisson model, Finland, 2006–2020

To identify seasonal trends, we plotted monthly iGAS, iGBS and iGCGS cases with moving averages. The individual graphs can be found in Supplementary Figures S1–S4. Moreover, we compared the monthly and yearly variation in incidence in a Poisson model and used it to predict monthly and yearly incidence trends (Figure 3). Further details on the calculated IRR are appended in Supplementary Table S3. As 2020 was an atypical year impacted by COVID-19 pandemic restrictions, we ran the model both including and excluding that year with comparable results. There were no major differences when running the model with the incidence adjusted for the number of days per month (results not shown).

Bars denote 95% confidence intervals.

In terms of monthly trends, for iGAS we observed a possible incidence peak in December and January, which was, however, not statistically significant (Figure 3A). There was a statistically significant dip in infections between August and November (Figure 3A). Conversely, iGBS and iGCGS peaked in July and August, with a dip in iGCGS infections in February (Figure 3B and C). See also Supplementary Figure S5, where we additionally provide the monthly incidence rate of iGBS per 100,000 population among individuals older than 1 year.

In terms of annual variation, 2012 was an atypical year for iGCGS infections, with fewer cases than expected. For iGAS and iGBS, there was a modest dip in incidence in 2011. Detailed results on the annual variation can be found in Supplementary Table S3 and Figures S1–S4.

## Discussion

Our registry-based study shows that the incidence of iGAS, iGBS (for individuals 45 years and older) and, in particular, iGCGS in Finland increased over the 15-year period of 2006 to 2020, peaked in 2018 and 2019 and then subsided in 2020. In 2020, COVID-19 pandemic restrictions were introduced, which greatly impacted the incidence of all infectious diseases with a person-to-person mode of transmission.

The mean annual incidence rate of iGAS was 4.1 (range: 2.1–6.7) per 100,000 population. This is in line with earlier reports, both from Finland [11-14] and worldwide [1,15-18]. However, our calculated incidence lies at the upper end of the reported spectrum, which reflects the increase in incidence over the study period, at a relative annual increase rate of 3%. A growing trend in iGAS in Finland has been noted previously [13,14]. Reports from other countries have been varying in this respect, with some studies reporting increasing [18] and others stable or even decreasing trends [15,19]. This discrepancy is probably due to the fluctuating nature of iGAS infections, characterised by epidemic waves, which renders their incidence and trends greatly dependent on the period under study.

Infections with iGBS occurred at a mean annual incidence rate of 5.2 per 100,000 (range: 4.0–6.3). This is approximately twice as high as in the years 1995 to 2000, despite a decreasing trend in iGBS among infants, an age group with a high incidence rate [20]. It is also higher than the mean incidence reported in a large multinational population study that included Finland, which identified an overall annual incidence of 3.4 per 100,000 persons between 2000 and 2010, with an increasing trend [2]. In our study, the increasing trend was limited to people 45 years and older. Improved perinatal screening practices of pregnant woman and intrapartum antibiotic prophylaxis for the GBS carriers seem to have been effective in preventing neonatal infections.

For iGCGS, we observed a sharply increasing trend (relative annual increase of 8%), with a mean annual incidence of 10.1 per 100,000. As a result of this marked upsurge, iGCGS infections surpassed those of iGAS and iGBS during the study period and constituted in 2020 the majority of invasive beta-haemolytic streptococcal infections in Finland. This upwards trend was noted previously in Finland in a small regional study by Rantala et al., covering the years 1995 to 2004 [21], but also worldwide [15,18,19,22-26]. This suggests that this is an ongoing global phenomenon rather than an isolated local trend. Our study showcased an even greater magnitude of incidence and increase than previous studies. It is not clear whether this difference is due to regional or surveillance differences or reflects an escalation in recent years, given that the large majority of available studies cover older time periods. Comparisons between studies are additionally complicated by methodological differences such as differences in case definitions (e.g. the type of clinical criteria and isolation sites included in the case definition) and/or focus on specific regional hospitals.

Overall, beta-haemolytic streptococcal infections seem to be increasing in Finland as well as worldwide. The reasons behind this are not clear and are likely to be multiple, encompassing both host and pathogen characteristics. For iGCGS, a partial explanation could be an improvement in diagnostic methods and microbial classification, as well as an increase in the frequency of performing blood cultures [27]. However, other than an increased frequency of blood cultures, there were no major changes in diagnostics and surveillance in Finland during our study period. The main explanation proposed is the vulnerability of a continuously ageing population with an increasing number of comorbidities [15,21]. This could be especially relevant for iGCGS infections, which overwhelmingly occur in patients with advanced age and underlying diseases [6,7]. It could likewise be the reason behind the rising trend in iGBS infections in the older age groups [2,28]. In terms of pathogen characteristics, increases in incidence could result from the emergence and spread of strains and clones with enhanced virulence. For example, iGAS epidemic clones are well described, and an association between specific *emm* types and severe iGCGS has been proposed as well [29,30].

Invasive beta-haemolytic streptococcal infections exhibited some seasonal patterns. For iGAS, we noted a statistically significant dip in infections in August and autumn, but no clear incidence peaks. Previous studies have observationally noted peaks in winter and early spring [15,19,31,32], and two Finnish studies additionally reported occasional mid-summer peaks [11,14]. Although our seasonal graphs showed a peak in December and January as well as occasional summer peaks, these were not statistically significant. Interestingly, the seasonal pattern for iGBS and iGCGS infections was opposite to that of iGAS, with peaks in July and August and a dip in iGCGS infections around February. Previous studies of iGBS seasonality have also observed summer peaks [33,34]. Few studies have looked at seasonal variation of iGCGS infections; however, two smaller-scale studies from Hungary and Norway observed summer peaks, supporting our results [15,19]. The contrasting seasonal patterns between iGAS and iGCGS are surprising and showcase that the notion that iGCGS resemble iGAS is simplistic.

We also noted differences between the Lancefield groups in terms of age and sex of cases, consistent with previous reports [7,11,12,15,16,32]. The median age of iGAS cases was lower than of iGBS and iGCGS cases. In addition, the age distribution differed between the three groups. The incidence of iGBS was particularly high among the infant population, which is not surprising, as neonates are a known risk group for perinatally acquired iGBS. In contrast, iGAS occurred across all ages. Characteristically, iGCGS showed a dual pattern, with only few cases among children and the vast majority of infections in the older age groups. Excluding infant iGBS cases, the incidence increased with age for all three Lancefield groups, with a particularly steep increase for iGCGS, especially for ages of 55 years and above. The incidence rate was overall higher in males compared with females for iGCGS and iGAS, whereas for iGBS, the association with sex was age group-dependent. Specifically, the incidence of iGBS was significantly higher among females compared with males in the age group 15–44 years. Pregnancy is a known risk factor for iGBS infections, which could explain this pattern, as this age range includes the peak reproductive years. In contrast, iGBS infections were significantly more common among males, in ages 55 years and above.

Interestingly, we noted a considerable number of repeat invasive infections with iGCGS and to a lesser extent also with iGBS. In contrast, repeat invasive infections with iGAS were rare. This difference has been noted earlier, but only in smaller studies, with fewer cases of recurrency [15,18,35,36]. Our study highlights the extent of iGCGS repeat infections, as well as the high number of episodes for certain individuals. It is unclear whether this feature is related to pathogen characteristics or to the structure of the patient population, given that iGCGS are prevalent among older individuals.

Invasive beta-haemolytic infections can be severe and potentially fatal. In our study, iGAS were associated with the highest mortality within the first week after diagnosis. Given the younger structure of the affected population, it is possible that iGAS are more aggressive than iGBS and iGCGS. In contrast, iGCGS appear to be less severe in younger ages; no deaths caused by GCGS were recorded among individuals younger than 30 years. Interestingly, at 30 days after notification, differences in mortality became less pronounced, and the CFR of iGCGS approached that of iGAS, at the level of 9–10%. The 30-day CFR of 9–10% is comparable to that reported for iGAS in earlier studies [12,14-17]; for iGCGS, due to the smaller scale of earlier studies, reported CFRs have been varying, ranging around 2–18% [7,15-17,21]. This CFR coupled with the high incidence demonstrated by our study suggests that the disease burden of iGCGS could be considerable and at the very least comparable to that of iGAS.

Despite the concerning observation of a continuously increasing incidence of beta-haemolytic streptococcal infections, systematic surveillance, especially for iGCGS, is lacking in many countries and at a European level. In many countries, iGCGS infections are not listed as notifiable diseases [37]. In Finland, surveillance of iGAS and iGBS is comprehensive at the national level; however, surveillance of iGCGS receives less attention and iGCGS isolates are not collected nor typed in a centralised manner.

Surveillance is further hampered by the grouping of isolates into Lancefield groups, even though groups overlap and comprise species with disparate features and pathogenicity [9]. The drawbacks of surveillance based on Lancefield groups are especially apparent for groups C and G, which are often pooled together, like in our study, because they are difficult to distinguish. A solution could be species-based notifications and surveillance. Another solution could be the systematic or targeted collection and standardised identification of invasive beta-haemolytic streptococcal isolates by a reference laboratory. This would also enable molecular typing, a critical piece of surveillance information lacking for iGCGS.

When interpreting our results, a number of limitations should be taken into account. Firstly, the Finnish case definition used here is solely laboratory-based and does not encompass isolations from sites beyond blood/CSF, nor clinical criteria as is the case in some other countries. By excluding clinical criteria, we may have missed deep tissue infections or cases where prompt antibiotic treatment contained the infection, precluding bacterial isolation from the specimen at the time of sampling. Secondly, we grouped iGCS and iGGS together, although they comprise diverse species, with possibly differing pathogenic properties. Finally, our register-based study did not include information on clinical presentations, treatment, underlying conditions or cause of death. On the other hand, our retrospective nationwide register-based study was based on an exceptionally comprehensive and large surveillance dataset, thanks to the mandatory notification of invasive streptococcal infections. 

## Conclusions

Our study has highlighted the need for enhanced attention to invasive beta-haemolytic streptococcal infections, which are becoming more frequent. Invasive iGCGS infections are of particular concern, due to inadequate surveillance and a lack of studies on this topic. Invasive iGAS and iGCGS diseases may be more dissimilar than previously thought, as they differ considerably in various respects, such as age–sex distribution, recurrence and seasonal patterns.

## Supplementary Data

1088000 Supplement
