# Supplementary material for: Invasive beta-haemolytic streptococcal infections, Finland, 2006 to 2020: increase in Lancefield group C/G infections
Source: Euro Surveill. 2023 Aug 3;28(31):2200807. doi: 10.2807/1560-7917.ES.2023.28.31.2200807 (PMC10401913; doi:10.2807/1560-7917.ES.2023.28.31.2200807)
Supplement: Supplement [file 22-00807_PASPALIARI_Strep_supplement.pdf]

SUPPLEMENTARY MATERIAL

This supplementary material is hosted by Eurosurveillance as supporting information alongside the article “Invasive beta-haemolytic streptococcal infections in Finland, 2006-2020: The rise of Lancefield group C/G infections”, on behalf of the authors, who remain responsible for the accuracy and appropriateness of the content. The same standards for ethics, copyright, attributions and permissions as for the article apply. Supplements are not edited by Eurosurveillance and the journal is not responsible for the maintenance of any links or email addresses provided therein.

Table S1. Annual relative incidence rate ratios (IRR) for iGAS, iGCGS and iGBS, with respective 95% confidence intervals (95% CI) and P-values, adjusted for age and sex. For iGBS, the annual relative incidence rate ratios are broken down by age group. Asterisks denote uncertainty due to small sample sizes.

	IRR	P-value	95% CI	
**iGAS**	1.03	<0.001	1.02	1.04
**iGCGS**	1.08	<0.001	1.07	1.09
**iGBS**	1.01	0.55	0.98	1.04
Age(y)				
0	0.92	<0.001	0.90	0.94
1-5	0.88*	0.369	0.65	1.17
6-14	0.68*	0.347	0.30	1.53
15-24	1.01	0.677	0.95	1.09
25-34	0.95	0.005	0.92	0.98
35-44	1.01	0.717	0.98	1.04
45-54	1.05	<0.001	1.03	1.07
55-64	1.03	0.003	1.01	1.05
65-74	1.05	<0.001	1.03	1.07
75-84	1.04	<0.001	1.02	1.06
>85	1.06	<0.001	1.04	1.09

Table S2. Adjusted incidence rate ratio (IRR) per age-group for males, females or both, for iGAS, iGBS and iGCGS, with respective 95% confidence intervals (95% CI) and P-values. Ratios are also adjusted for year of isolation and are calculated with age-group 25-34 as reference. For iGBS, the incidence patterns per age group differed considerably per sex, and we, therefore, decided to only show each sex individually. The last column shows the adjusted incidence rate ratio of males to females. Asterisks denote uncertainty due to small sample sizes.

**iGAS**															
	Males				Females				Both sexes			Males:Females			
**Age (y)**	**IRR**	**P-value**	**95% CI**		**IRR**	**P-value**	**95% CI**		**IRR**	**95% CI**		**IRR**	**P-value**	**95% CI**	
**0**	1.40	0.210	0.83	2.39	0.53	0.073	0.27	1.06	0.89	0.52	1.26	1.81	0.441	0.26	1.79
**1-5**	0.67	0.041	0.46	0.98	0.37	<0.001	0.25	0.56	0.49	0.36	0.63	1.24	0.382	0.76	2.02
**6-14**	0.46	<0.001	0.33	0.66	0.22	<0.001	0.15	0.32	0.32	0.23	0.40	1.46	0.103	0.93	2.30
**15-24**	0.55	<0.001	0.40	0.76	0.43	<0.001	0.32	0.58	0.48	0.37	0.59	0.88	0.482	0.62	1.25
**25-34**	ref				ref				ref			0.69	0.006	0.52	0.90
**35-44**	1.69	<0.001	1.29	2.20	0.97	0.809	0.75	1.26	1.26	1.03	1.49	1.19	0.170	0.93	1.54
**45-54**	1.69	<0.001	1.29	2.20	0.76	0.044	0.58	0.99	1.14	0.92	1.35	1.52	0.002	1.17	1.97
**55-64**	2.09	<0.001	1.62	2.71	0.96	0.772	0.75	1.24	1.42	1.16	1.68	1.49	0.001	1.17	1.90
**65-74**	2.55	<0.001	1.97	3.32	1.17	0.239	0.90	1.51	1.73	1.41	2.05	1.50	0.001	1.17	1.92
**75-84**	3.34	<0.001	2.54	4.40	1.78	<0.001	1.38	2.31	2.42	1.96	2.87	1.28	0.057	0.99	1.66
**>85**	4.55	<0.001	3.27	6.34	2.83	<0.001	2.16	3.70	3.53	2.77	4.29	1.10	0.554	0.79	1.54
**All**												1.25	.	1.10	1.39

**iGBS**												
	Males				Females				Males:Females			
**Age (y)**	**IRR**	**P-value**	**95% CI**		**IRR**	**P-value**	**95% CI**		**IRR**	**P-value**	**95% CI**	
**0**	154.23	<0.001	95.90	248.06	29.56	<0.001	21.58	40.50	0.90	0.229	0.76	1.07
**1-5**	0.00*	0.998	0.00	.	0.04*	<0.001	0.01	0.14	.*	.	.	.
**6-14**	0.04*	0.002	0.00	0.30	0.00*	0.998	0.00	.	.*	.	.	.
**15-24**	0.26	0.003	0.11	0.64	0.19	<0.001	0.11	0.34	0.24	<0.001	0.11	0.50
**25-34**	ref				ref				0.17	<0.001	0.11	0.26
**35-44**	2.89	<0.001	1.71	4.90	0.69	0.052	0.47	1.00	0.73	0.017	0.60	0.94
**45-54**	3.94	<0.001	2.38	6.51	0.72	0.073	0.51	1.03	0.94	0.543	0.77	1.15
**55-64**	8.34	<0.001	5.16	13.47	1.06	0.734	0.76	1.47	1.36	<0.001	1.16	1.59
**65-74**	12.14	<0.001	7.52	19.59	1.56	0.007	1.13	2.16	1.34	<0.001	1.17	1.53
**75-84**	23.07	<0.001	14.31	37.18	3.01	<0.001	2.19	4.13	1.32	<0.001	1.15	1.52
**>85**	32.66	<0.001	19.64	54.30	4.52	<0.001	3.20	6.38	1.24	0.017	1.04	1.49

**iGCGS**															
	Males				Females				Both sexes			Males:Females			
**Age (y)**	**IRR**	**P-value**	**95% CI**		**IRR**	**P-value**	**95% CI**		**IRR**	**95% CI**		**IRR**	**P-value**	**95% CI**	
**0**	1.23	0.626	0.53	2.86	1.12	0.776	0.51	2.44	1.17	0.50	1.84	0.82	0.723	0.27	2.45
**1-5**	0.15	<0.001	0.06	0.43	0.12	<0.001	0.04	0.33	0.13	0.04	0.23	0.96	0.950	0.24	3.83
**6-14**	0.10	<0.001	0.04	0.26	0.08	<0.001	0.03	0.20	0.09	0.03	0.15	0.96	0.944	0.28	3.31
**15-24**	0.35	<0.001	0.21	0.58	0.37	<0.001	0.24	0.58	0.36	0.24	0.48	0.70	0.239	0.39	1.26
**25-34**	ref				ref				ref			0.74	0.092	0.53	1.05
**35-44**	2.42	<0.001	1.78	3.29	1.21	0.236	0.88	1.65	1.72	1.35	2.10	1.49	0.004	1.14	1.96
**45-54**	5.42	<0.001	4.09	7.20	3.07	<0.001	2.36	4.01	4.08	3.29	4.86	1.31	0.003	1.10	1.58
**55-64**	13.66	<0.001	10.42	17.92	5.12	<0.001	3.97	6.60	8.77	7.15	10.38	1.99	<0.001	1.73	2.29
**65-74**	27.11	<0.001	20.73	35.46	9.88	<0.001	7.71	12.65	17.23	14.10	20.36	2.04	<0.001	1.81	2.31
**75-84**	49.92	<0.001	38.15	65.32	21.89	<0.001	17.15	27.93	33.85	27.72	39.98	1.70	<0.001	1.51	1.91
**>85**	84.05	<0.001	63.75	110.80	40.60	<0.001	31.77	51.89	59.14	48.19	70.10	1.54	<0.001	1.35	1.77
**All**												1.63	.	1.51	1.76

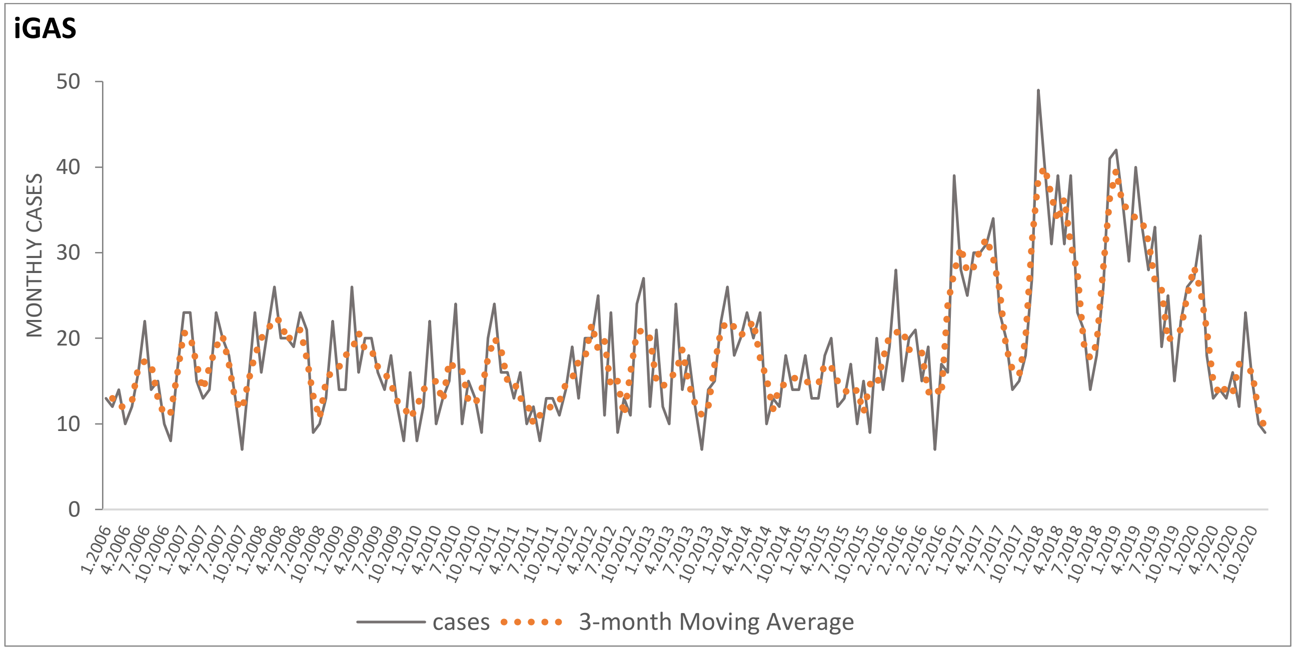

Figure S1. Monthly cases of iGAS, with 3-month moving average, Finland, 2006-2020.

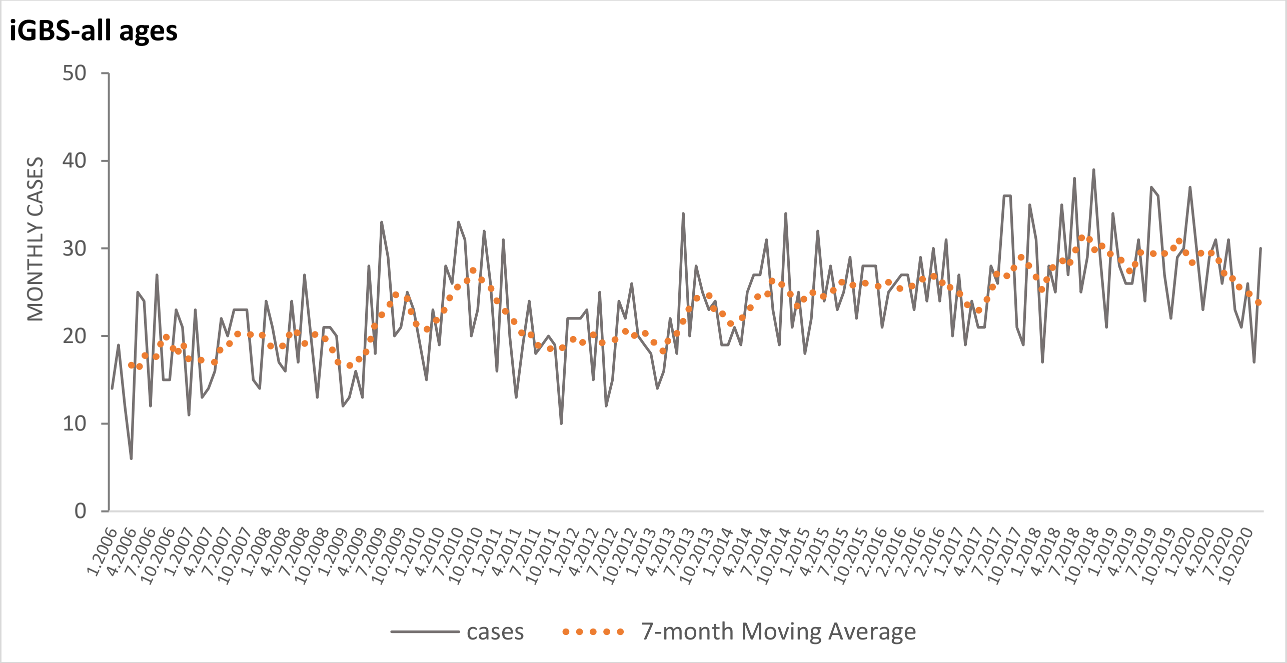

Figure S2. Monthly cases of iGBS with 7-month moving average, Finland, 2006-2020.

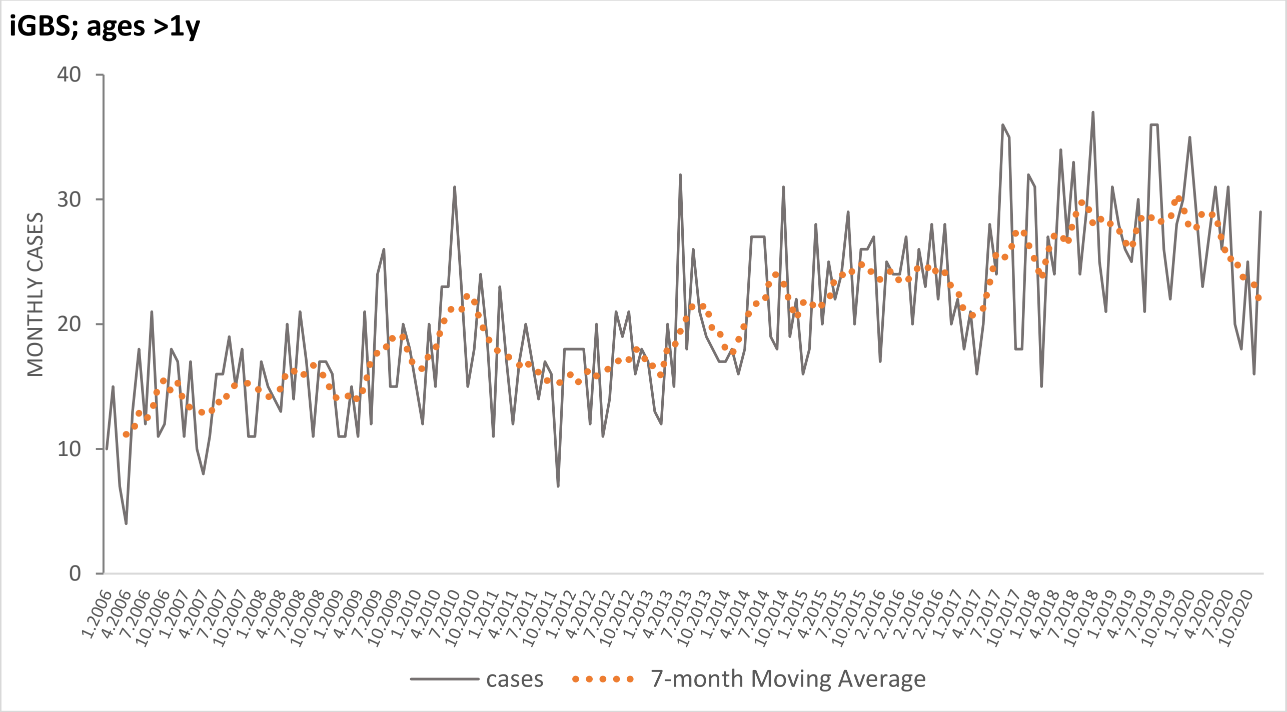

Figure S3. Monthly cases of iGBS among individuals aged >1 y, with 7-month moving average, Finland, 2006-2020.

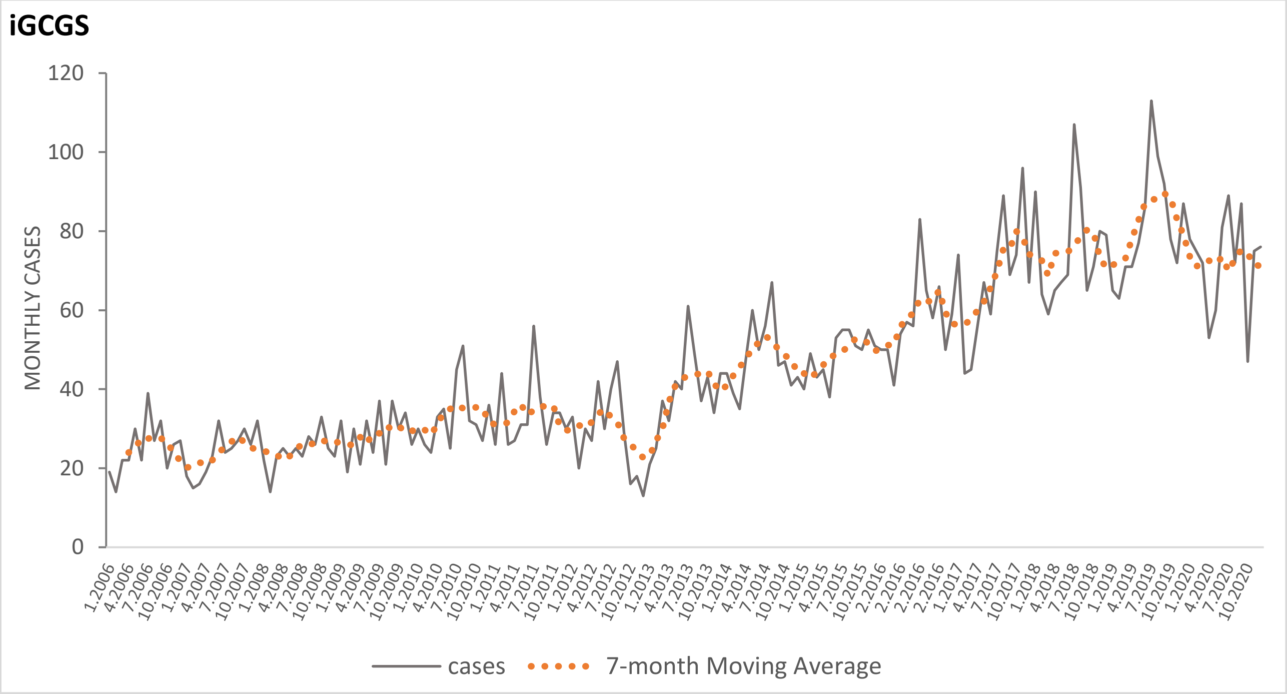

Figure S4. Monthly cases of iGCGS with 7-month moving average, Finland, 2006-2020.

Table S3. Incidence rate ratios (IRR) per calendar month and calendar year, adjusted for calendar year and month, respectively, for iGAS, iGBS (all ages and ages >1 y) and iGCGS, with respective 95% CI and P-values. The month of January and the year 2006 are set as references. Years included are 2006-2020 ("including 2020") or 2006-2019 ("excluding 2020").

**iGAS**								
	including 2020				excluding 2020			
**Month**	**IRR**	**P-value**	**95% CI**		**IRR**	**P-value**	**95% CI**	
**January**	ref				ref			
**February**	0.95	0.480	0.81	1.10	0.92	0.343	0.79	1.09
**March**	0.96	0.611	0.82	1.12	0.99	0.871	0.84	1.16
**April**	0.87	0.085	0.74	1.02	0.91	0.23	0.77	1.07
**May**	0.94	0.431	0.81	1.10	0.98	0.776	0.83	1.15
**June**	0.89	0.164	0.77	1.05	0.93	0.388	0.79	1.09
**July**	0.87	0.093	0.75	1.02	0.90	0.199	0.76	1.06
**August**	0.68	<0.001	0.58	0.81	0.70	<0.001	0.59	0.84
**September**	0.62	<0.001	0.53	0.74	0.60	<0.001	0.50	0.73
**October**	0.61	<0.001	0.51	0.73	0.61	<0.001	0.51	0.74
**November**	0.65	<0.001	0.55	0.78	0.68	<0.001	0.57	0.81
**December**	1.01	0.908	0.87	1.17	1.07	0.404	0.91	1.25
**Year**								
**2006**	ref							
**2007**	1.26	0.028	1.02	1.55				
**2008**	1.24	0.041	1.01	1.51				
**2009**	1.08	0.444	0.88	1.34				
**2010**	0.95	0.653	0.77	1.18				
**2011**	0.92	0.429	0.74	1.14				
**2012**	1.18	0.109	0.96	1.45				
**2013**	0.99	0.926	0.80	1.22				
**2014**	1.15	0.182	0.94	1.41				
**2015**	0.97	0.748	0.78	1.19				
**2016**	1.24	0.037	1.01	1.51				
**2017**	1.58	<0.001	1.31	1.92				
**2018**	2.00	<0.001	1.66	2.40				
**2019**	1.87	<0.001	1.55	2.25				
**2020**	1.08	0.459	0.88	1.33				
**iGBS age≥1y**								
	including 2020				excluding 2020			

Month	IRR	P-value	95% CI		IRR	P-value	95% CI	
**January**	ref				ref			
**February**	0.99	0.865	0.83	1.16	1.01	0.928	0.84	1.20
**March**	1.00	0.966	0.84	1.18	1.05	0.622	0.88	1.25
**April**	0.89	0.192	0.75	1.06	0.91	0.308	0.76	1.09
**May**	1.20	0.027	1.02	1.40	1.24	0.012	1.05	1.47
**June**	1.14	0.12	0.97	1.33	1.19	0.042	1.01	1.41
**July**	1.27	0.003	1.08	1.48	1.32	0.001	1.12	1.56
**August**	1.27	0.003	1.08	1.48	1.37	<0.001	1.16	1.61
**September**	1.07	0.429	0.91	1.26	1.15	0.116	0.97	1.36
**October**	1.14	0.120	0.97	1.33	1.20	0.038	1.01	1.42
**November**	1.04	0.615	0.89	1.23	1.13	0.174	0.95	1.34
**December**	1.13	0.140	0.96	1.33	1.17	0.068	0.99	1.39
**Year**								
**2006**	ref							
**2007**	1.02	0.884	0.82	1.27				
**2008**	1.11	0.348	0.90	1.37				
**2009**	1.14	0.216	0.93	1.41				
**2010**	1.36	0.003	1.11	1.66				
**2011**	1.07	0.531	0.87	1.32				
**2012**	1.16	0.159	0.94	1.43				
**2013**	1.28	0.018	1.04	1.57				
**2014**	1.45	<0.001	1.19	1.76				
**2015**	1.56	<0.001	1.29	1.90				
**2016**	1.57	<0.001	1.29	1.90				
**2017**	1.59	<0.001	1.31	1.93				
**2018**	1.80	<0.001	1.49	2.18				
**2019**	1.86	<0.001	1.54	2.25				
**2020**	1.70	<0.001	1.40	2.06				
**iGBS all ages**								
	including 2020				excluding 2020			
**Month**	**IRR**	**P-value**	**95% CI**		**IRR**	**P-value**	**95% CI**	
**January**	ref				ref			
**February**	0.99	0.906	0.85	1.16	1.01	0.868	0.86	1.19
**March**	0.98	0.843	0.84	1.15	1.03	0.709	0.88	1.21
**April**	0.91	0.260	0.78	1.07	0.93	0.395	0.79	1.10
**May**	1.19	0.024	1.02	1.37	1.23	0.009	1.05	1.44

**June**	1.10	0.219	0.95	1.28	1.15	0.083	0.98	1.35
**July**	1.22	0.007	1.06	1.42	1.27	0.002	1.09	1.49
**August**	1.23	0.006	1.06	1.42	1.31	0.001	1.12	1.53
**September**	1.06	0.461	0.91	1.23	1.12	0.156	0.96	1.32
**October**	1.13	0.110	0.97	1.31	1.19	0.034	1.01	1.39
**November**	1.07	0.416	0.91	1.24	1.14	0.098	0.98	1.34
**December**	1.09	0.249	0.94	1.27	1.13	0.134	0.96	1.32
**Year**								
**2006**	ref							
**2007**	1.00	0.969	0.83	1.21				
**2008**	1.03	0.754	0.86	1.24				
**2009**	1.07	0.483	0.89	1.28				
**2010**	1.25	0.013	1.05	1.49				
**2011**	0.97	0.748	0.81	1.17				
**2012**	1.02	0.801	0.85	1.23				
**2013**	1.09	0.373	0.91	1.30				
**2014**	1.21	0.038	1.01	1.44				
**2015**	1.27	0.008	1.06	1.51				
**2016**	1.26	0.010	1.06	1.50				
**2017**	1.28	0.006	1.07	1.52				
**2018**	1.40	<0.001	1.18	1.67				
**2019**	1.43	<0.001	1.20	1.69				
**2020**	1.32	0.002	1.11	1.57				
**iGCGS**								
	including 2020				excluding 2020			
**Month**	**IRR**	**P-value**	**95% CI**		**IRR**	**P-value**	**95% CI**	
**January**	ref				ref			
**February**	0.87	0.020	0.78	0.98	0.86	0.016	0.76	0.97
**March**	0.89	0.051	0.80	1.00	0.89	0.058	0.79	1.00
**April**	0.93	0.212	0.83	1.04	0.97	0.568	0.86	1.09
**May**	1.07	0.249	0.96	1.19	1.11	0.082	0.99	1.24
**June**	1.06	0.260	0.96	1.18	1.07	0.266	0.95	1.20
**July**	1.41	<0.001	1.27	1.56	1.44	<0.001	1.30	1.61
**August**	1.28	<0.001	1.16	1.42	1.33	<0.001	1.20	1.49
**September**	1.11	0.051	1.00	1.24	1.11	0.068	0.99	1.25
**October**	1.04	0.440	0.94	1.16	1.10	0.087	0.99	1.24
**November**	1.08	0.163	0.97	1.20	1.10	0.116	0.98	1.23
**December**	1.08	0.163	0.97	1.20	1.09	0.123	0.98	1.23

**Year**					
**2006**	ref				
**2007**	0.94	0.473	0.80	1.11	
**2008**	0.88	0.120	0.75	1.03	
**2009**	1.04	0.657	0.89	1.21	
**2010**	1.19	0.025	1.02	1.38	
**2011**	1.20	0.016	1.03	1.40	
**2012**	1.02	0.767	0.88	1.19	
**2013**	1.37	<0.001	1.19	1.59	
**2014**	1.69	<0.001	1.47	1.95	
**2015**	1.71	<0.001	1.49	1.97	
**2016**	2.00	<0.001	1.75	2.29	
**2017**	2.36	<0.001	2.07	2.70	
**2018**	2.63	<0.001	2.31	3.00	
**2019**	2.82	<0.001	2.48	3.21	
**2020**	2.50	<0.001	2.19	2.85	

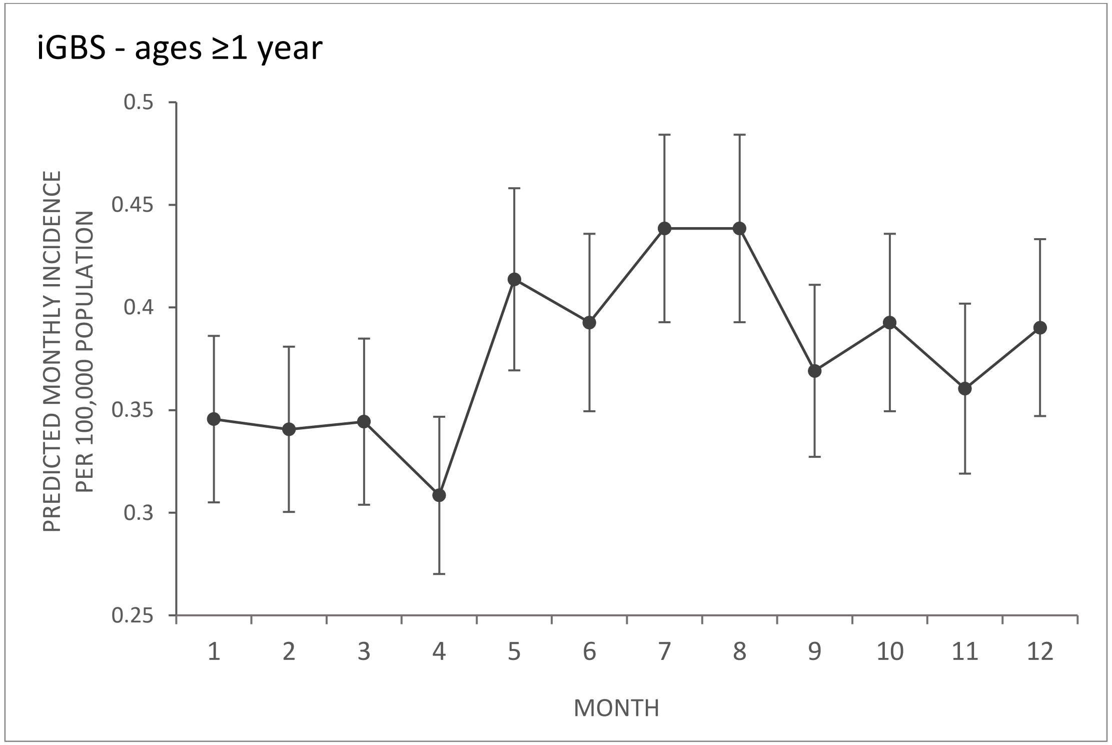

Fig. S5. Monthly incidence rate of iGBS per 100,000 population among individuals aged >1y, as predicted by a Poisson model. Bars denote 95% CI.
